# Utility of video-fundoscopy and prospects of portable stereo-photography of the ocular fundus in neurological patients

**DOI:** 10.1186/s12883-022-02578-5

**Published:** 2022-02-19

**Authors:** Tigran Khachatryan, Tahseen Mozaffar, Lilit Mnatsakanyan

**Affiliations:** 1 Department of Neurology, University of California, Irvine, USA; 2 Department of Pathology & Laboratory Medicine, University of California, Irvine, USA

**Keywords:** Fundoscopy, Ophthalmoscope, Smartphone, 3D, Fundus photography

## Abstract

**Background:**

Proper evaluation of ocular fundi is an integral part of neurological examination. Unfortunately, neurology residents are increasingly uncomfortable performing fundoscopy and interpreting findings because of diminishing skills and lack of experience. This became more prominent during the COVID-19 pandemic as fundoscopy requires proximity to the patient.

With the recent dramatic improvement of smartphone cameras, fundus photography using the PanOptic Ophthalmoscope (Welch Allyn, Skaneateles Falls, NY) with a smartphone adapter offered an alternative to direct fundoscopic examination. We present the first experience with our own design of a universal smartphone adapter.

**Methods:**

This is a single-center case series, consecutive for a single user and certain presenting neurological symptoms, which is aimed to evaluate the feasibility and practicality of a new, universal PanOptic smartphone adapter. Presenting symptoms included headache, ocular symptoms, seizure, or encephalopathy. We used 3D modeling and printing techniques to create the adapter.

We also developed a methodology of capturing stereoscopic images of the optic disc using a single smartphone camera, but the method was not systematically evaluated in this paper.

**Results:**

Here we present our initial experience of fundus video/photography in patients, who presented with encephalopathy, headache, seizure, vision loss, and other ocular symptoms. Fundoscopic abnormalities were discovered in 11 out of 100 patients. Some were incidental findings and were unrelated to the presentation. In one case, fundoscopy played a critical role in establishing the correct diagnosis.

**Conclusions:**

Our custom-designed smartphone adapter allowed obtaining high-quality video and photo recordings using PanOptic Ophthalmoscope. The acquisition of high-quality photos enables a high-yield diagnostic tool and allows revisiting the image in the patient’s chart. Improvement of smartphone cameras opens vast horizons for stereo-fundoscopy and 3D reconstruction of the ocular fundus without using sophisticated and costly equipment. Microscopic eye movements allow taking snapshots of two side-by-side photos for 3D reconstruction and stereoscopic image viewing, which is the next level of optic disc assessment.

## Background

Fundoscopic examination is a basic competency requirement for all Neurologists and an important component of neurological examination [1, 2]. Traditional direct fundoscopy requires that examiners position themselves close to the patient which became a serious limitation during COVID-19 pandemic [3]. As part of a resident quality improvement project, medical records of 100 consecutive patients, admitted to the Department of Neurology at UC Irvine Medical Center during the COVID-19 pandemic, were retrospectively reviewed. None of the patients underwent a fundoscopic examination.

With dramatic improvement of camera quality in modern smartphones, fundus photography using PanOptic Ophthalmoscope (Welch Allyn, Skaneateles Falls, NY) with a smartphone adapter offered an alternative to direct examination of ocular fundus. We explored the idea of creating our very own design of universal smartphone adapter for PanOptic Ophthalmoscope to fit all smartphone types, which would help reducing very close contact with the patient and reduce the time needed for examination. We focused on PanOptic ophthalmoscope due to widespread availability, ease of use and large field of view.

Stereo fundoscopy has been validated as an effective method of optic nerve head assessment and is widely used in ophthalmological practice for diagnosis and treatment of glaucoma and diabetic retinopathy [4–7]. To the best of our knowledge, there are no studies evaluating stereo fundoscopy in the emergency room setting for evaluation of neurological disorders, as the required equipment is not portable and not readily available in Emergency Departments.

## Methods

This study is a prospective, single-center case series aimed to evaluate the feasibility and practicality of a new PanOptic smartphone adapter, which was conducted as part of resident quality improvement project at UC Irvine Medical Center. Cases are consecutive for a single user and certain presenting symptoms as detailed below. Thus, it is not a true prevalence study. All methods were performed in accordance with the relevant guidelines and regulations.

**Fig. 1:**
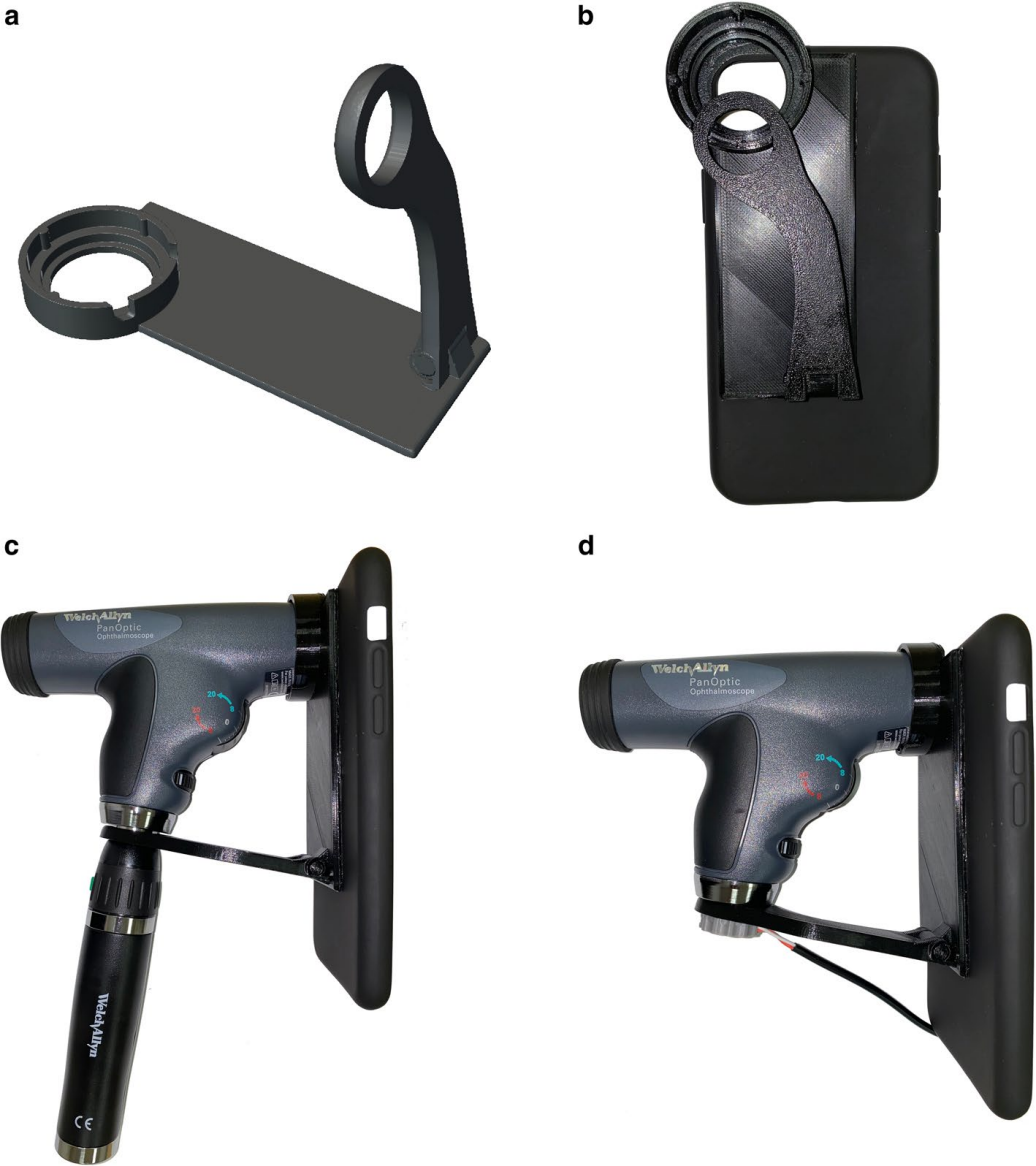
Universal smartphone adapter for Panoptic ophthalmoscope. **a**: 3D model of the adapter. **b**: The adapter is permanently attached to the smartphone case. **c**: Assembled ophthalmoscope with factory battery. **d**: assembled ophthalmoscope with smartphone-powered light source

The adapter was designed using MeshMixer (Autodesk Inc.) 3D modeling software (Fig. 1-a). It was then printed with Polyethylene terephthalate glycol filament on a 3D Fused Deposition Modeling printer.

The attachment consists of a foldable arm, a flat surface with double-sided adhesive and an ocular piece, which fits exactly the ocular part of PanOptic Ophthalmoscope (Fig. 1-c). The adapted can be permanently attached to any smartphone case and, with the foldable arm, can be seamlessly carried in the pocket (Fig. 1-b). When fully assembled, our adapter resembles iExaminer (Welch Allyn Inc., Skaneateles Falls, New York, USA) adapter but has several advantages, including foldable arm, low profile and compatibility with all smartphones. Additionally, we designed a smartphone powered light source, which eliminated the need for charging and carrying the battery piece (Fig. 1-d). Thus, one needs to carry only the head of PanOptic ophthalmoscope to obtain fundoscopic images.

Patients, presenting to the emergency department with complaints of headache, ocular symptoms (visual disturbances, blindness, diplopia, pain), seizures or encephalopathy underwent fundoscopic videography and/or photography. We did not use pharmacological pupillary dilation for any of the patients. In cooperative patients, the examiner instructed the patient to focus gaze on a stationary object and sequentially obtained video recordings from both eyes. In uncooperative patients the bedside nurse carefully held the patient’s eyelid open while the examiner was obtaining the recordings.

Images were captured using iPhone 11 Pro (Apple Inc., CA, USA) smartphone. Photographs were directly uploaded to patient’s chart on Epic using Haiku app for IOS (Epic systems corporation, Verona, WI). Video data was de-identified and deleted after subtracting all necessary snapshots. Fundus videographs and photographs were then reviewed by on-call senior resident and later by the attending neurologist on service during formal rounds. Reviewers were not blinded to prior reviews due to importance of ongoing resident teaching and feedback on interpretation skills. Therefore, we planned to analyze and report outcomes as specific case illustrations rather than period prevalence, quality or accuracy measures.

**Fig. 2:**
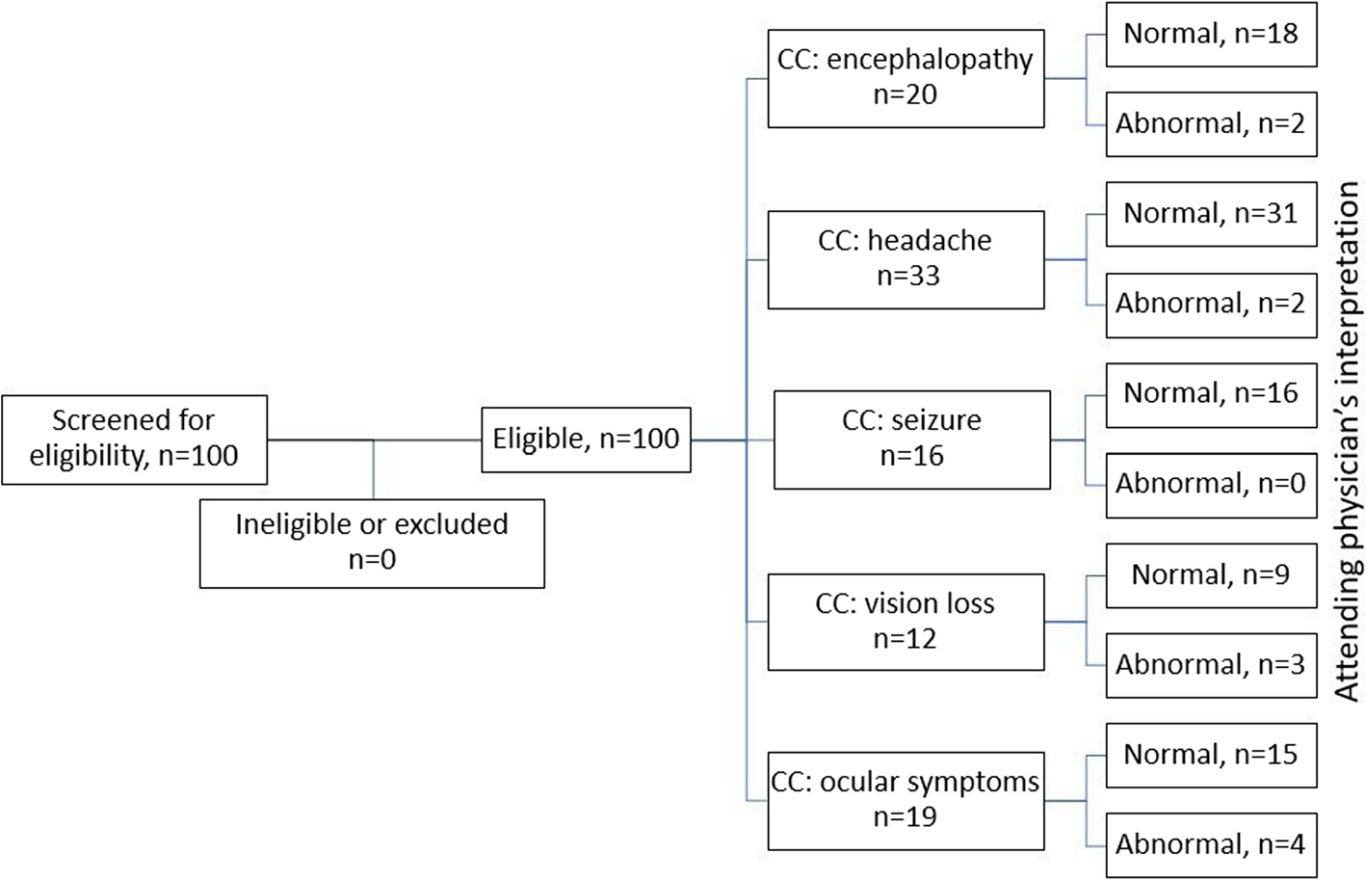
Strengthening the Reporting of Observational Studies in Epidemiology (STROBE) flowchart (CC – chief complaint)

Data is reported in accordance with Strengthening the Reporting of Observational Studies in Epidemiology (STROBE) Statement (Fig. 2), [8].

We also developed a methodology of capturing stereoscopic images of the optic disc using a single smartphone camera. Healthy volunteer subjects were asked to look sequentially at two objects or light sources located 6 ft away from the eye and 30 cm apart from each other. Video fundoscopy was performed while the subject was performing the task. The camera remained static in respect to the subject’s forehead, but eyeball moved in the horizontal plane, providing visualization of the fundus under two different angles equal to ten degrees. Subsequently, snapshots from each respective position were obtained, which essentially represented a pair of stereoscopic images. Google cardboard or similar 3D headset was utilized to view stereoscopic images. The aforementioned methodology of stereoscopic imaging was developed relatively late in the study, thus was not routinely utilized in presented group of patients.

Upon admission to the hospital, all patients or their legal representatives signed admission terms and conditions agreement, which includes statements about obtaining medical video/photographs. Furthermore, fundoscopy is a part of neurological exam and does not require separate written consent. There are no identifiable features in the presented group of photographs.

## Results

Data was analyzed after performing fundus video/photographs on 100 patients over the period from December 2019 to February 2021. None of the patients or legal representatives declined enrollment. The presentation included encephalopathy in 20%, headache in 33%, seizure in 16%, vision loss in 12% and other ocular symptoms (diplopia, decreased acuity of vision, eye pain) in 19% of patients. The mean age of the examinees was 44.7 years (range 20–89).

**Fig. 3:**
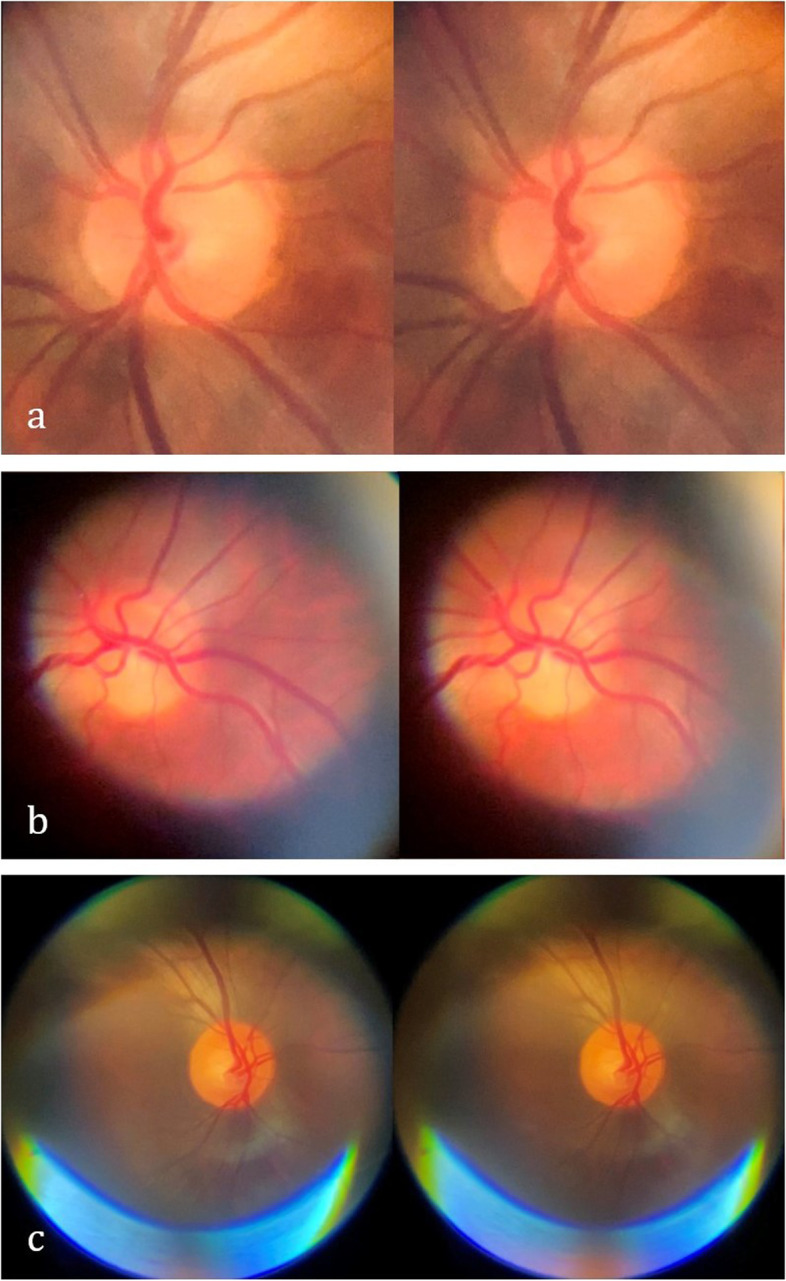
Stereoscopic images of the ocular fundi generated by capturing two consecutive frames from a video recording after subtle eye movement (requires google cardboard or similar for stereoscopic viewing)

**Fig. 4:**
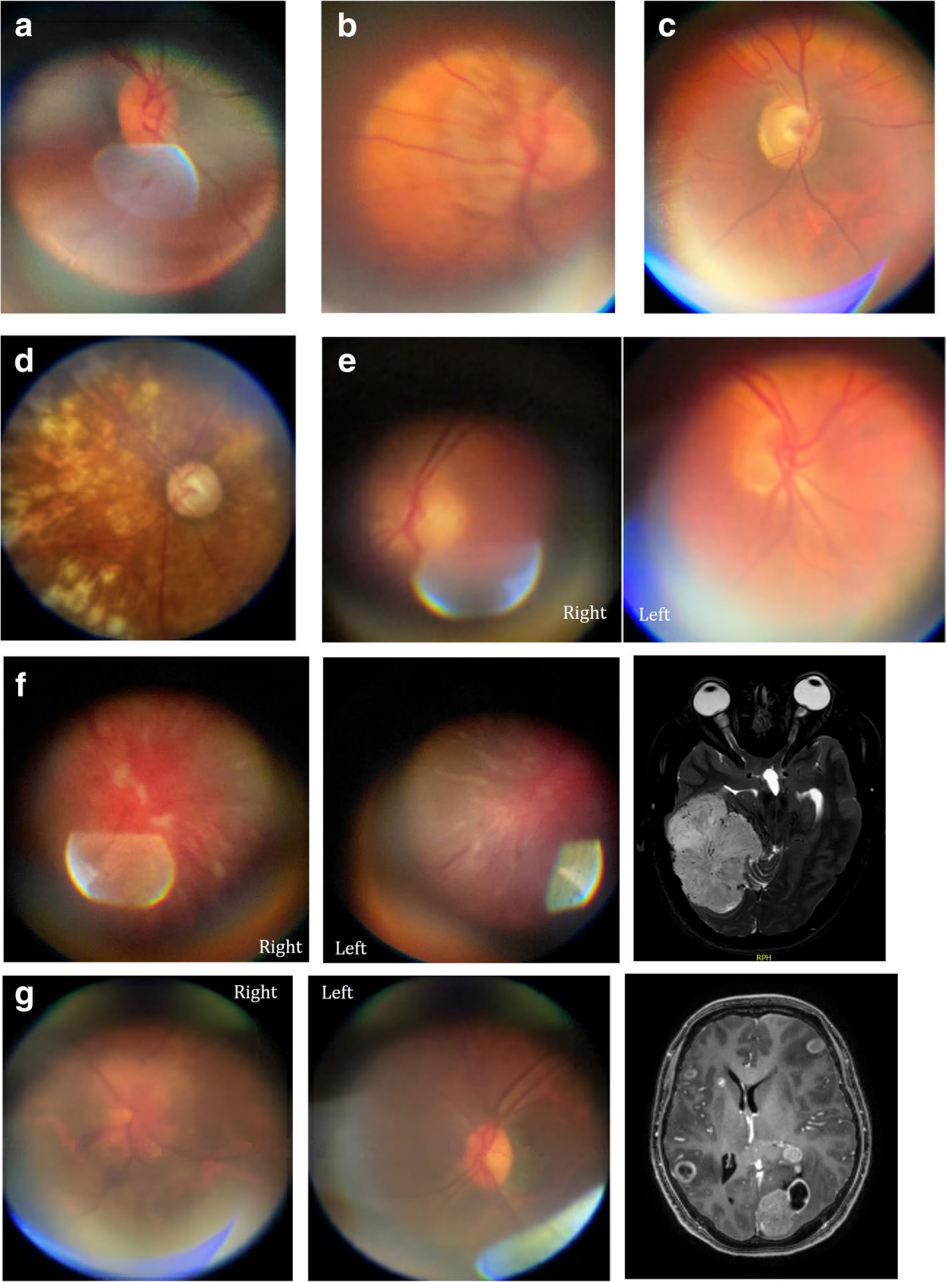
Fundoscopic abnormalities and incidental findings. **a**. Incidental finding of a small optic disc in a 57 yo woman with blurry vision and migraine attack. **b**. Excessive myelination of optic disc in an 89 yo woman with binocular diplopia of unclear etiology **c**. Optic disc pallor in a 30 yo woman with vision loss, who was subsequently diagnosed with Neuromyelitis Optica. **d**. Multiple laser ablation scars in a 76 yo man with blurred vision, fixed and dilated pupil, who was found to have acute angle closure glaucoma. **e**. Bilateral grade 2 papilledema in a 55 yo with Anaplastic ependymoma. **f**. Bilateral grade 4 papilledema in a 40 yo woman with blurry vision, who was found to have a large right tentorial meningioma. **g**. Foster-Kennedy syndrome in a 50 yo man with metastatic lung cancer.

**Fig. 5:**
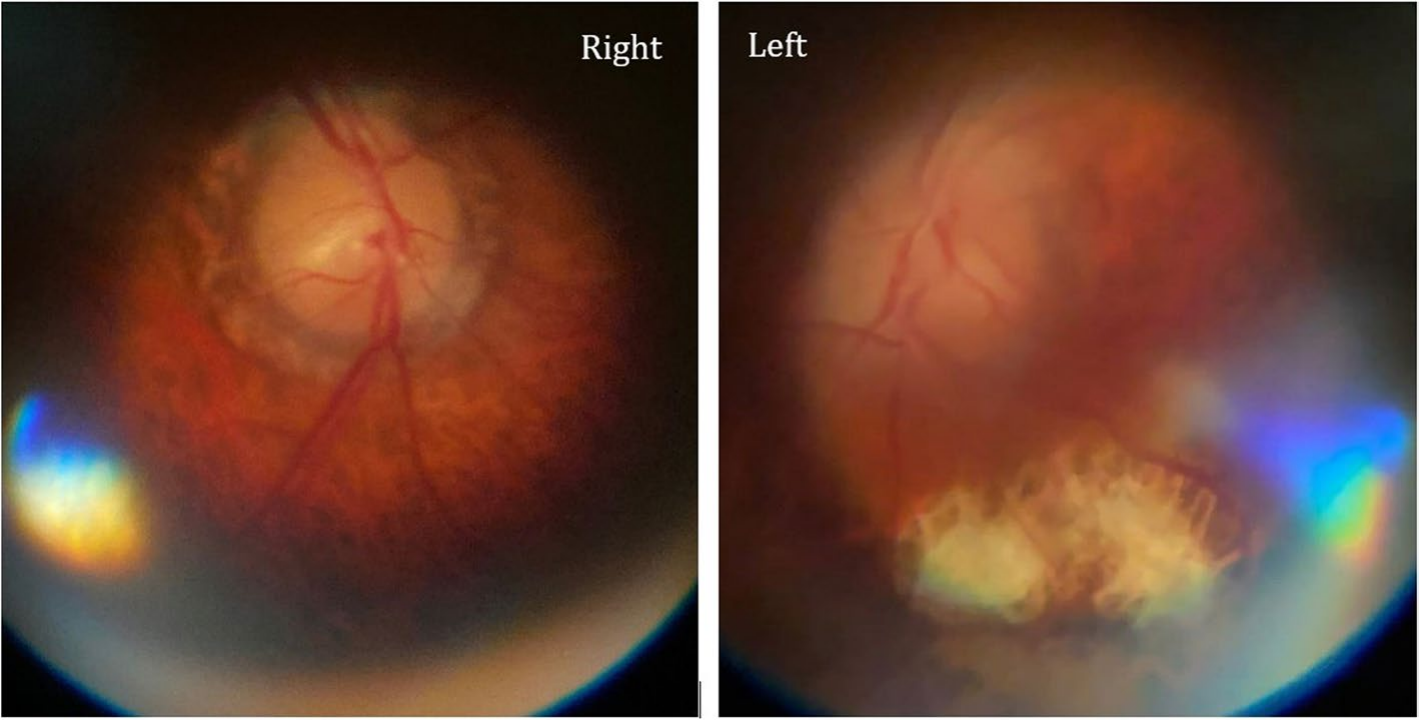
Bilateral papilledema (grade 1 on the right and grade 3 on the left) in a 78-years-old patient with leptomeningeal carcinomatosis

Stereoscopic images were obtained on two healthy volunteers (Fig. 3a and b), and one patient with normal fundoscopic findings. Stereoscopic images resulted in subjectively better perception of cup-to-disc ratio and relation of vessels to the optic disc when compared to 2D images acquired from the same patient (Fig. 3c). Fundoscopic abnormalities were discovered in 11 patients, although some were incidental findings as these were unrelated to the presentation or chief complaint (Fig. 4 a-g). In one patient, fundoscopy played a critical role in establishing the diagnosis. As an example of the clinical utility of fundus photography using PanOptic ophthalmoscope, we present a case of a78 yo man with history of uncontrolled diabetes and chemotherapy due to gastric adenocarcinoma was brought in by family due to altered mental status and decreased oral intake. On initial evaluation patient was diagnosed with diabetic keto acidosis due to beta hydroxybutyrate level of > 4.0 mmol/L, bicarbonate level of 19 mmol/L and anion gap of 16 mmol/L prompting admission to medical ICU for further care. Neurology was consulted due to history of bilateral lower extremity weakness after a chiropractic manipulation. Fundoscopy was performed as part of the neurological examination and showed bilateral grade 1 papilledema on the right and grade 3 papilledema on the left (Fig. 5). Based on the findings lumbar puncture was recommended which revealed opening pressure of 55 cm H2O and 235 nucleated cells. Final cytological diagnosis of leptomeningeal carcinomatosis due to gastric adenocarcinoma was confirmed by CDX2 immunostaining. Patient underwent VP shunt placement and was discharged to acute rehabilitation facility with outpatient follow-up.

## Discussion

Since first direct visualization of ocular fundus by Hermann von Helmholtz in 1851, fundoscopy has become an integral part of neurological examination [9–11]. Many authors have emphasized the importance of proper fundoscopic examination in neurological patients [11, 12]. However, with introduction of neuro-imaging modalities such as CT and MRI, non-ophthalmology specialists are having an increasingly difficult time to perform fundoscopy or interpret the findings because of diminishing skills and lack of experience [9, 11–14]. This is partly explained by the necessity to dilate the pupil for adequate examination, which inherently makes serial neurological examinations more difficult. In 1886, Jackman and Webster published the first human fundus photograph, beginning a new era in visualization of ocular fundus [10, 14, 15]. With advancement of technology, non-mydriatic fundus cameras came into practice [9, 16]. The wide field of view and possibility of performing the examination without pharmacologic dilation of the pupil became a huge advantage of non-mydriatic fundus cameras [9, 17]. It has a number of other advantages, including higher rate of successful visualization, possibility to share the image among multiple providers, avoiding repeated examinations causing patient discomfort as well as storing images for comparison in the future [9, 12, 21, 22]. Several studies have shown that images even can be obtained by non-medical personnel after a short training period, unlike direct fundoscopy, when visualization and interpretation occur simultaneously [2, 3, 10, 12, 16].

However, there is a host of limitations to non-mydriatic fundus photography. Even handheld non-mydriatic cameras are bulky and difficult to carry around at all times and are not widely used outside the ophthalmology clinic settings [23]. The cost of such cameras is another limitation of its universal use, particularly in countries with limited resources [12]. Additionally, direct ophthalmoscopy allows visualization of retinal venous pulsation, while such interpretation is impossible by viewing still photographs [24]. Finally, trying to time the good fundoscopic view and capture a photo is a skill, that may take longer to acquire than performing a direct fundoscopy [23].

In 2001 PanOptic (Welch Allyn Inc., Skaneateles Falls, New York, USA) introduced its new ophthalmoscope and later developed an FDA approved smartphone adapter called iExaminer [9, 23, 25]. According to the manufacturer’s website, the adapter is suitable only for iPhone 4, 4S, 6, 6S, 6 Plus and 6S Plus which are outdated models and unfortunately do not provide satisfactory image quality. Therefore, we designed our own adapter which is suitable for any smartphone. Thus, it does not have to be redesigned with each new generation of smartphones. As of the time of this writing, the adapter is not FDA approved and is meant to be used only in research settings. Current smartphones can take high-quality video recordings, allowing the examiner to rewind the video and choose the best frame for storing in the patient’s chart. It also allows assessing the venous pulsation. What is more exciting, microscopic eye movements allow extracting side-by-side photos from the source fundoscopic video, which can be used for 3D reconstruction and stereoscopic image viewing, which has been shown to increase diagnostic accuracy in ophthalmological pathologies (Fig. 4 a-c) [4–7, 18].

In our study image acquisition was successful in all patients, even those who were uncooperative or had depressed level of consciousness, mainly due to utilization of video recording and ability to use less light intensity thanks to high-quality of current smartphone cameras. There was a steep learning curve for examining difficult patients, which sometimes required an assistant to keep eyelids open.

Multiple studies have shown that medical students and junior residents prefer using fundus photography over direct ophthalmoscopy and it results in greater diagnostic accuracy [10, 15, 19, 20]. In our experience, medical students rotating with the neurology department were able to take high-quality fundus videographs after only 15–20 min of demonstration and practice.

We are planning to evaluate the new PanOptic adapter further by including all residents in the department. We will also expand its use to all inpatient and outpatient clinical settings and evaluate limiting factors for widespread utilization of fundus video/photography, such as hesitation to adopt a new exam tool or medico-legal concerns tied to image interpretation.

## Conclusions

We present our initial experience of feasibility and practicality of a custom-built universal smartphone adapter for PanOptic ophthalmoscope to obtain videographs and photographs of ocular fundus in patients presenting to the emergency department with a neurological complaint. Acquisition of high-quality photos enables a high-yield diagnostic tool, allows revisiting the image in patient’s chart and serves as a great teaching tool. This study was intended to test the feasibility and practicality of smartphone fundoscopy but not diagnostic accuracy, hence the limitation of the study is absence of a control group and blinded outcome.

Improvement of smartphone cameras, along with the method of obtaining stereoscopic images as detailed in the article, creates an opportunity to investigate the utility of stereo-fundoscopy in daily clinical practice without using sophisticated and costly equipment.

## Data Availability

The datasets used and/or analyzed during the current study are available from the corresponding author on reasonable request.
